# Developing a Capsule Clinic—A 24-Hour Institution for Improving Primary Health Care Accessibility: Evidence From China

**DOI:** 10.2196/41212

**Published:** 2023-01-09

**Authors:** Dongliang Li, Rujia Zhang, Chun Chen, Yunyun Huang, Xiaoyi Wang, Qingren Yang, Xuebo Zhu, Xiangyang Zhang, Mo Hao, Liming Shui

**Affiliations:** 1 School of Public Health Fudan University Shanghai China; 2 School of Public Health and Management Wenzhou Medical University Wenzhou China; 3 Engineering Research Center of Intelligent Medicine (2016E10011) The First Affiliated Hospital of Wenzhou Medical University Wenzhou China; 4 Yinzhou District Health Bureau Ningbo China

**Keywords:** primary health care, accessibility, capsule clinic, 24-hour clinic, big-data, China, United Nations, internet clinic

## Abstract

Telehealth is an effective combination of medical service and intelligent technology. It can improve the problem of remote access to medical care. However, an imbalance in the allocation of health resources still occurs. People spend more time and money to access higher-quality services, which results in inequitable access to primary health care (PHC). At the same time, patients’ usage of telehealth services is limited by the equipment and their own knowledge, and the PHC service suffers from low usage efficiency and lack of service supply. Therefore, improving PHC accessibility is crucial to narrowing the global health care coverage gap and maintaining health equity. In recent years, China has explored several new approaches to improve PHC accessibility. One such approach is the capsule clinic, an emerging institution that represents an upgraded version of the internet hospital. In coordination with the United Nations, the Yinzhou district of Ningbo city in Zhejiang, China, has been testing this new model since 2020. As of October 2022, the number of applications in Ningbo was 15, and the number of users reached 12,219. Unlike internet hospitals, the entire process—from diagnosis to prescription services—can be completed at the capsule clinic. The 24-hour telehealth service could also solve transportation problems and save time for users. Big data analysis can accurately identify regional populations’ PHC service needs and improve efficiency in health resource allocation. The user-friendly, low-cost, and easily accessible telehealth model is of great significance. Installation of capsule clinics would improve PHC accessibility and resolve the uneven distribution of health resources to promote health equity.

## Background

Combining intelligent technology with health care has become increasingly popular [1,2]. Telehealth is the remote provision of clinical health care and health administration services via information and telecommunication technologies [3]. In exploring suitable methods to meet people’s increasing demands, countries are establishing intelligent medical models in telehealth to provide convenient health services [4] and solve geographical, temporal, and economic problems of accessibility to health care. Doctors use telehealth to transmit digital imaging, conduct video consultations, and make medical diagnoses. Telehealth began in the 20th century [5], with the advent of television, and has developed rapidly in the 21st century along with advancements in technology. Most people currently have access to basic devices, such as mobile phones and computers, which can be used to obtain telehealth services. With improved accessibility of health care through telehealth, individuals in rural areas and busy urban areas can often connect more easily with a provider. In the United States and the United Kingdom [6], telehealth is a popular trend. According to the National Health Service Long Term Plan in the United Kingdom, “digitally enabled care will go mainstream” [7].

Compared with other countries, China is a late starter in the field of telehealth. In recent years, China has been committed to applying internet technology to medical services. The internet hospital is a rapidly developing, new telehealth model that is gaining wide popularity [8]. The establishment of internet hospitals could help some citizens overcome temporal and geographical barriers to accessing traditional medical services [9]. However, the issue of accessibility to primary health care (PHC) services has not been completely resolved. Owing to urban-rural differences and unbalanced allocation of regional health human resources [10], the phenomenon of inequality in health resource allocation still exists [11]. Furthermore, as the population continues to age [12] and the number of chronic diseases grows [13], the demand for health services increases every year. Researchers have found that accessibility of PHC services has been challenged by insufficient funds [14], limited-service locations [15], poverty-stricken areas, and inconvenient transportation [16-18]. The use of PHC is inefficient because people rely on doctors in higher-tier hospitals [19]. Although the internet hospital is a promising public health tool because it could significantly increase access to health care for medically underserved populations, some obstacles remain. For example, people with low digital information literacy, especially older people and children, cannot easily use internet health care. Low digital literacy remains the key barrier to accessing intelligent health systems [20,21]. In order to use telehealth, such as internet hospitals, mobile devices have high requirements [22,23]. Inadequate communication facilities hinder telehealth services [24,25] for many people in remote areas, and many older people experience barriers to using smartphones. Internet hospitals must improve applications for older adults. In addition, patients also face the problem of waiting for drug delivery after receiving remote consultation services via the internet hospital, which could cause a loss of time [26,27]. Moreover, big data is not being used well in the allocation of drug resources; the advantages of big data analysis of population characteristics and rational allocation of drug resources cannot be realized in internet hospitals.

In many cases, although internet hospitals address the problem of unbalanced distribution of health resources, the shortcomings mentioned above must be addressed to provide access to PHC services. In the near future, the application of telehealth will not only increase convenience and access to health care but also reduce costs. Telehealth services should also be more user-friendly and implement low-cost devices to facilitate remote health care services. Intelligent medical service systems should be better integrated with the traditional medical models, and internet hospitals must be upgraded to meet the needs of all members of society.

## Capsule Clinics in China

The accessibility of PHC services in China is usually considered in terms of geographical accessibility [28-30], economic accessibility, and temporal accessibility [31,32]. To that end, we propose the development of the Internet Hospital 2.0 in the era of big data, in the context of Ningbo, a city in China with an estimated population of over 9 million individuals [33]. Ningbo residents face challenges in accessing PHC services. They remain disappointed with the inconvenient primary care model and are anxious about the time and economic costs of daily medical visits, particularly during the COVID-19 pandemic. Ningbo is a coastal city that includes many islands and other remote areas. People living in remote villages or islands experience greater challenges in accessing and paying for PHC services.

In an attempt to combine the use of available public health data with the provision of easy access to health care, and toward the achievement of universal health coverage, the World Health Organization implemented a pilot project for developing a higher-quality and efficient medical and health service e-system. The Yinzhou district in Ningbo is one of the pilot locations. Yinzhou has created a specialized health service model in the Chinese context—the capsule clinic, an upgraded version of the internet hospital. The capsule clinic (20 m^2^ in area) is a novel type of health service facility that began operating in 2020. It incorporates traditional medical models, including diagnosis and prescription. Capsule clinic services comprise 3 parts: health examination, consultation, and an intelligent pharmacy.

The special clinic, which relies on Zhejiang province’s excellence in digital reform and Yinzhou’s superior health care resources, is an emerging form of medical treatment facility that can provide residents with more convenient PHC services than those currently available. One advantage of advanced intelligent technology–based models is that they increase geographical accessibility to PHC. Capsule clinics are placed within communities to enable residents’ convenient access to PHC without leaving the community. The capsule clinic offers some important advantages, such as 24-hour services, comprehensive medical services that include the entire process from diagnosis to prescription services, and efficient allocation of health resource services. The capsule clinic overcomes some of the administrative shortcomings of the internet hospital.

**Figure 1:**
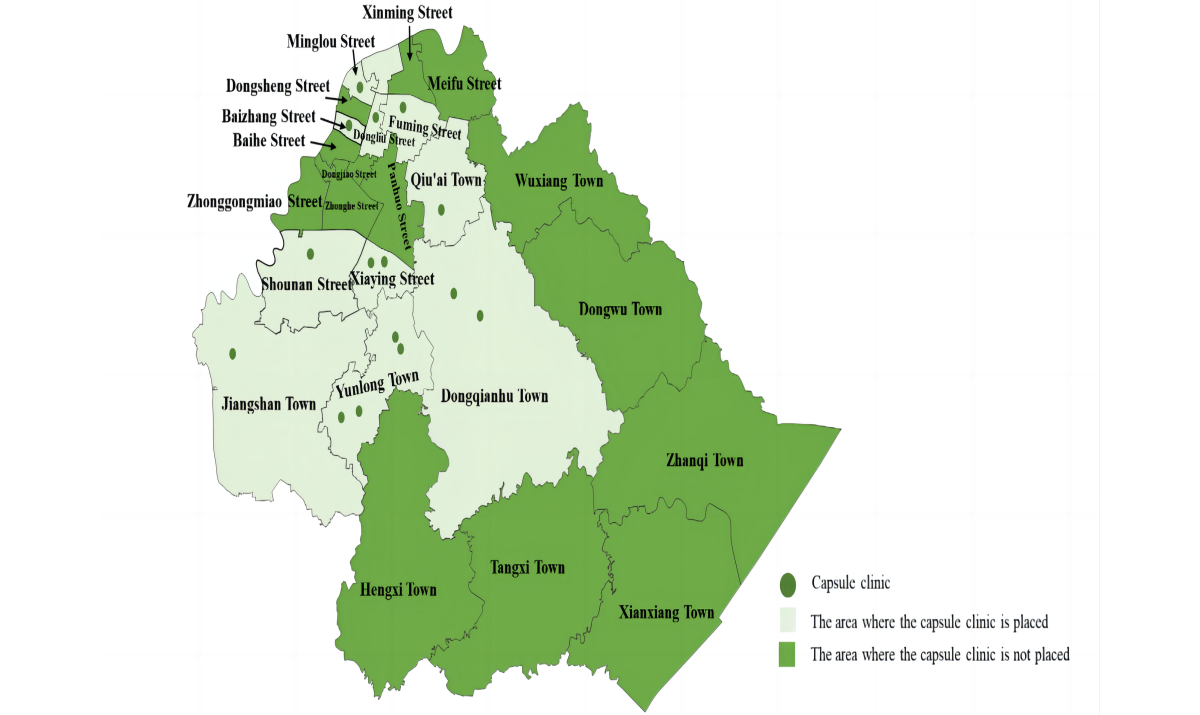
Distribution of capsule clinics in Yinzhou.

In recent years, Zhejiang has attached great importance to the combination of intelligent technology and medical services, allowing grassroots residents to enjoy higher-quality and inclusive PHC services. Figure 1 shows that as of October 2022, there were 15 applications for capsule clinics in Yinzhou (Figure 1). According to China’s “one village, one health care room” principle, Yinzhou plans to establish over 190 capsule clinics across communities or villages and expand throughout China.

## The Function of the Capsule Clinic

**Figure 2:**
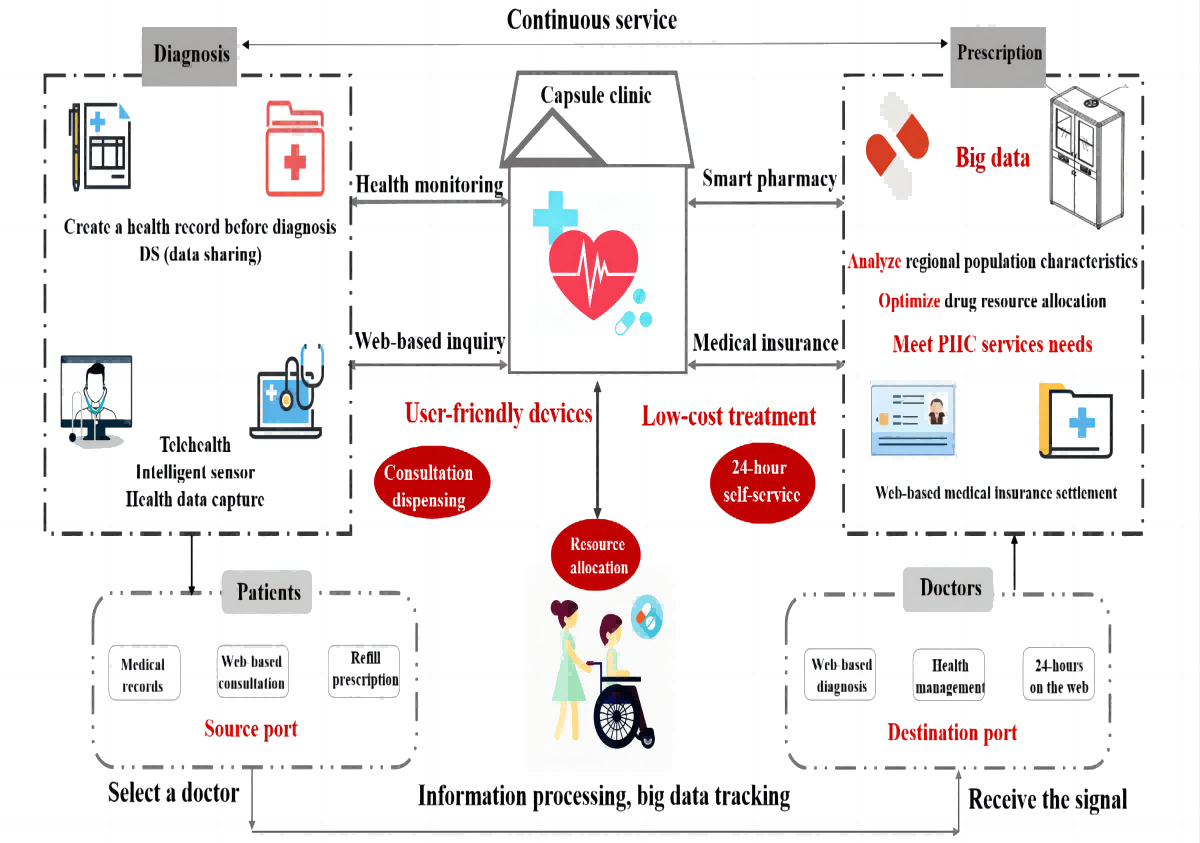
Main functions of the capsule clinic. PHC: primary health care.

Figure 2 shows that several critical hardware and software components are included in the capsule clinic to ensure the necessary functions to meet the medical demands of residents living nearby. The capsule clinic has continuous medical services, including diagnosis and prescription, which fit the traditional medical model. Doctors can communicate with patients for remote health care consultations around the clock. Patients can access services on their own any time they need them and even gain access to essential medicine. In addition, patients can use the telehealth service of capsule clinics to consult doctors from higher-tier hospitals; this feature provides an opportunity for PHC service resources to be equitably deployed, especially for people in remote areas.

The capsule clinic, a product of the digital reform, has developed rapidly and is devoted to improving geographic accessibility to PHC. For each community, the capsule clinic will be conveniently located within 15 minutes of the community’s health service center, which is responsible for the full scope of the capsule clinic. At nearby capsule clinics, people located in remote areas (mountainous areas and islands) can access health resources equal to those available to urban residents. This measure is intended to benefit the public and solve issues of not only geographic accessibility but also temporal accessibility; with the capsule clinic, people do not have to miss work or travel long distances for medical appointments.

Although some internet hospitals and telehealth centers are also available 24 hours a day, capsule clinics are more helpful to residents because they provide comprehensive services. In the past, patients received their medications within a few days after receiving a web-based diagnosis. The capsule clinic takes advantage of intelligent technology to help residents save time in receiving their medications. After completing a remote consultation with the doctor, patients can easily access the capsule clinic’s intelligent pharmacy to pick up their medication. They can also refill prescriptions from offline doctors. Moreover, if further examination or tests are needed, patients can visit the higher-tier hospitals within the period determined by the capsule clinic doctors. If hospitalization is required, treatment can be transferred to the hospital, as determined by the capsule clinic doctors. The capsule system embodies the advantages of an integrated health care delivery system. It facilitates convenient access to health care and makes good use of PHC resources.

Big data analysis and artificial intelligence can assist doctors in a variety of ways, such as by detecting lesions and improving diagnostic efficiency. They also play a role in improving medical services and easing constraints on medical resources. With the deep integration of digital technology and medical measures, as well as comprehensive improvements in patients’ health literacy, diversified medical and health service models can provide convenience for doctors and patients, thus improving the efficiency of diagnosis and treatment. Big data allows for systematic analysis of the relevant characteristics and medical needs of people in each region and the subsequent reasonable allocation of drug resources. For instance, in a community with a wide distribution of older or chronic patient populations, the capsule clinic would be equipped with more drugs to treat geriatric issues and chronic diseases. One benefit of a big data–based system is the reduction in medical processing time and distance, which significantly improves the efficiency and quality of the medical care provided and facilitates optimum resource allocation.

## Comparing the Capsule Clinic With the Traditional Hospital and Internet Hospital

### Overview

The capsule clinic has the physical appearance and essential equipment of a traditional medical institution. It also includes 24-hour remote consultation, integrated consultation and pharmacy services, optimal health resource allocation, and health management functions. The clinic has the strong support and oversight of a traditional hospital, and offers web-based services for relatively simple problems that do not necessitate a visit to the hospital.

**Figure 3:**
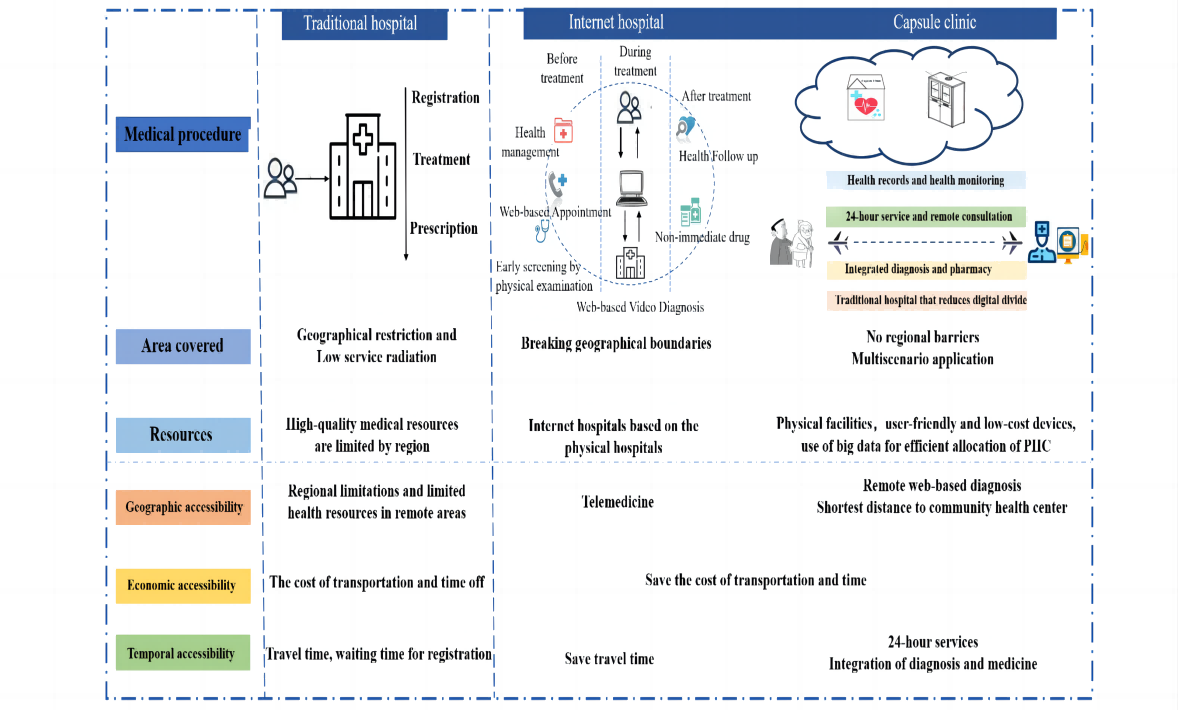
Comparison of the 3 medical models. PHC: primary health care.

Digital technology will soon be deeply integrated with medical treatment and will provide important support for medical professionals’ diagnosis and treatment decisions. However, the internet hospital model [34,35] still has much room for improvement, for example, in terms of medical insurance and the allocation of medical resources. With the support of big data technology, capsule clinics analyze the regional group characteristics, realize the reasonable allocation of essential drug resources, and meet the health needs of different populations. Even more importantly, capsule clinics may alleviate shortages in human resources chronically faced by primary-level medical institutions. The capsule clinic allows community residents to conveniently purchase medication 24 hours a day, taking pressure off in-person facilities. Remote consultation can also reduce pressures on grassroots medical staff and, thus, improve work efficiency. Figure 3 provides comparison of 3 medical models.

In order to understand the user experience of the capsule clinic during the implementation process, we conducted the research during November and December 2021. The research team visited communities and villages where capsule clinics were established, such as Haichuang Community, Lijia Village in Yunlong Town, and Dongfu Community in Qianhu Street, to conduct qualitative interviews with users (Table 1). Qualitative interviews collected basic user characteristics, home addresses, user acceptance of the capsule clinic, and feelings about using it. The interviews revealed that internet hospitals are different from capsule clinics, which have physical clinics and equipment, and rely on medical resources from offline hospitals. Users can not only experience the same diagnosis and prescription services in capsule clinics as in offline hospitals, but also use intelligent equipment that improves the accessibility of PHC services. As capsule clinics are physical entities, patients view them as more reliable than intangible internet hospitals. Moreover, this physicality makes them more user-friendly and greatly reduces use disparities caused by the digital divide [36-38].

**Table 1:** Qualitative interviews of the user experience of capsule clinics.

Interviewee	Personal information				Interview content
	Age (years)	Gender	Address of capsule clinic	Job	
Interviewee 1	45	Female	Haichuang Community Health Service Station, Yinzhou District, Ningbo	Employee of a state-owned enterprise	“It is time-saving for me to go to the clinic after work. It is the same as the local community hospital and I can find a doctor remotely through the capsule clinic whenever I need.”
Interviewee 2	28	Male	Hefeng Community Health Service Station, Hefeng Creative Plaza, Yinzhou District, Ningbo	Employee of a foreign company	“I've used capsule clinics accessible by walking. Compared with the Internet hospital, the capsule clinic can give me the feeling of being treated in a physical hospital. I could complete the procedure of diagnosis and treatment and did not have to wait for drugs.”
Interviewee 3	50	Male	Shanglijia Village Committee, Yunlong Town, Yinzhou District, Ningbo	Factory worker	“The capsule clinic is in our village, it's like a convenience store, and I can go in and take my blood pressure when I'm walking by. I come here to take my blood pressure and use the screening program every day.”
Interviewee 4	68	Female	Zhongxing Community Health Service Station, Dongliu Street, Yinzhou District, Ningbo	Retiree	“I haven't been to the capsule clinic but I've heard about it because I'm too old to use these smart devices.”
Interviewee 5	38	Female	Party Mass Service Center of Junrui Community, Xiaying Street, Yinzhou District, Ningbo	Full-time housewife	“The capsule clinic is closer, which is more convenient than the community hospital. The common drugs that my children and older adults need can be dispensed here under the consultation of the doctor.”
Interviewee 6	35	Male	Guanying Village Committee, Yunlong Town, Yinzhou District, Ningbo	Private entrepreneur	“I like the 24-hour service of the capsule clinic, which is convenient and fast.”

### Operation Process of the Capsule Clinic

**Figure 4:**
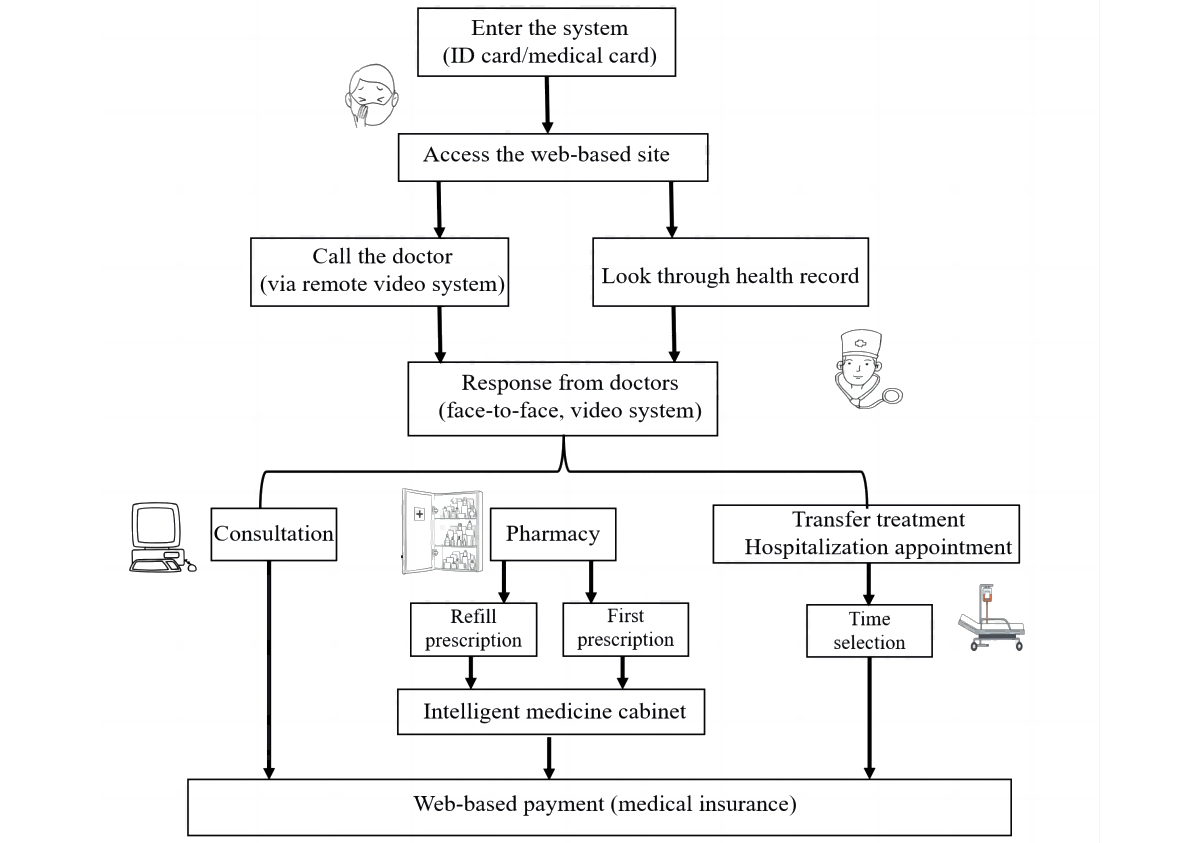
Flowchart of capsule clinic health care services for patients.

**Figure 5:**
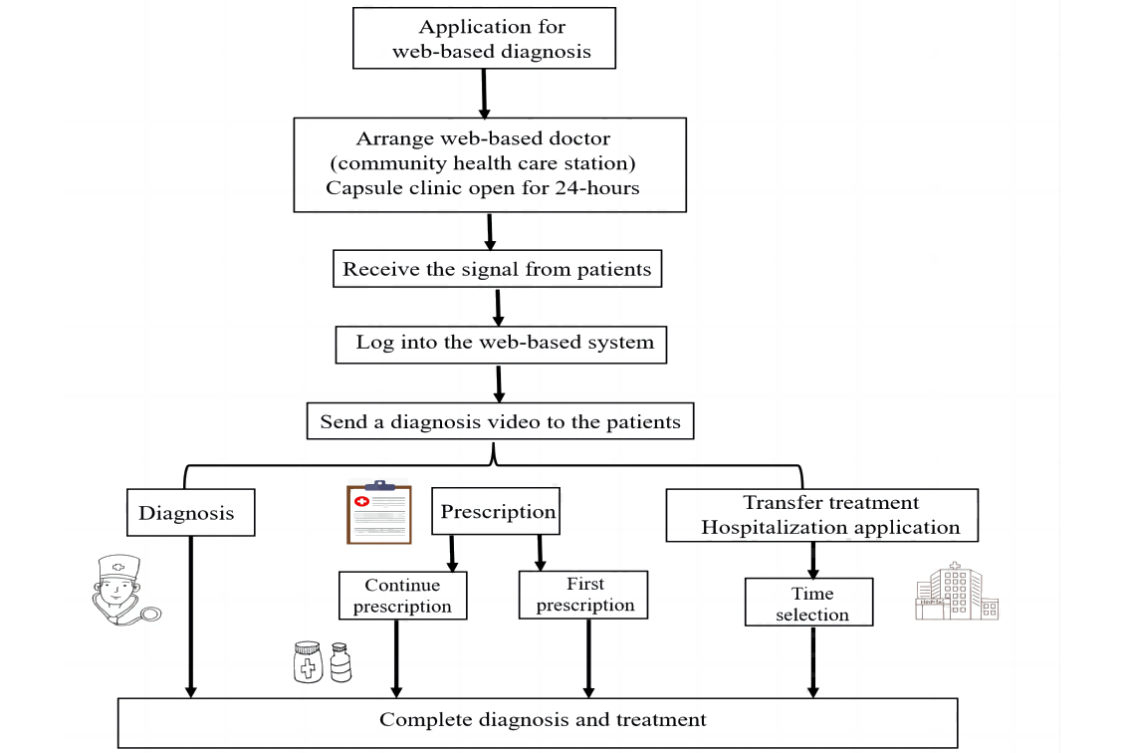
Flowchart of capsule clinic health care services for doctors.

The patient arrives at the capsule clinic and enters. They scan their identification or insurance card to gain access to the web-based site, where they can review their medical records, refill prescriptions, and schedule a remote appointment with a doctor. Figure 4 illustrates this flowchart of capsule clinic health care services for patients. After receiving the signal that a patient needs a consultation, a doctor from a higher-tier medical institution provides web-based medical services, including face-to-face consultation via a remote video system. Figure 5 illustrates this flowchart of capsule clinic health care services for doctors. As noted, patient prescriptions are generally filled immediately after the web-based consultation. The big data system collects the characteristics of the population in the area where the capsule clinic is located, along with past medication habits. It configures the drug resources in the intelligent pharmacy in accordance with the provided information and the doctor’s advice. The available drug resources are configured to meet the needs of more than 90% of the population. Patient prescriptions are provided by an intelligent pharmacy that focuses on the community residents’ demands and their physical conditions. Furthermore, if residents only want to refill medications at the capsule clinic, they can receive the same prescription with the help of web-based doctors.

Unlike the internet hospital, the capsule clinic not only provides consultation services but also supports patients’ medication needs with the intelligent medicine cabinet under the approval of the offline physical clinic. This measure provides great convenience for patients with long-term drug demands due to chronic diseases. When patients complete a physical hospital visit and have qualified for web-based prescription refills, they can pick up their next refill at the capsule clinic. If patients are identified as needing further examination or hospitalization during the remote consultation, the doctor will schedule an appointment for them at the parent hospital.

The number of capsule clinic users reached 12,219 by October 2022 (Table 2). Since 2020, the number of users has shown a gradual upward trend. Most of these patients are aged between 18 and 60 years; older adults have problems using the capsule clinics owing to the digital divide. Regarding the choice of drug purchased at the capsule clinic, 94.09% of patients buy Rx (Receptor X) medication, whereas only 4.49% choose over-the-counter medication, and 1.42% visit the capsule clinic simply to use the health monitoring program. This shows that most patients use both diagnosis and treatment and medicine dispensing functions in capsule clinics. In terms of the total cost of related drugs, more than 80% of patients spent less than 300 Chinese Yuan (US $43.20) per visit. Perhaps most of the PHC needs of nearby residents can be met by the capsule clinics, where people can avail of basic diagnostic services and buy essential medicines.

Various data from the past 3 years show that the number of capsule clinics in use is increasing year by year. Residents who live nearby are curious about the new medical institutions and may try the health monitoring program when walking past. Users are very interested in the 24-hour service provided by the clinic. In addition, the low number of users may have resulted from inadequate publicity; therefore, many people, especially older adults, are afraid to try this novel medical model. Although older adults still experience obstacles to using the new intelligent medical model, the use of capsule clinics is on the rise owing to the rapid development of internet technology, the expansion of internet access facility coverage, and the facilitation of using facilities. The gap between patients’ “willingness to use” and “ability to use” capsule clinics may influence the digital divide.

Thus far, based on the geographical distribution mentioned above, the capsule clinics are distributed in each residential location, which satisfies the residents’ desire to avail of PHC services close to home and at any time. Compared with the distance that the residents previously had to travel to reach community hospitals, the geographic accessibility of PHC services has been largely addressed by the capsule clinics. Furthermore, the issue of economic accessibility has also been addressed as people with jobs can avoid the cost of absence for medical treatment and the cost of transportation. With the help of big data, the capsule clinic’s smart pharmacy could estimate and allocate drugs in accordance with residents’ medication characteristics, meet residents’ medication needs, and achieve good coverage of PHC services.

**Table 2:** Characteristics of patients of capsule clinics from 2020 to 2022.

Characteristics		Patients, n (%)			
		2020	2021	2022	Total
Total users		2392 (19.58)	4595 (37.60)	5232 (42.82)	12,219 (100)
**Age (years)**					
	≤18	430 (17.98)	879 (19.13)	925 (17.68)	2234 (18.28)
	18-40	874 (36.54)	1923 (41.85)	1740 (33.25)	4537 (37.13)
	40-60	739 (30.89)	1168 (25.42)	1650 (31.54)	3557 (29.11)
	≥60	349 (14.59)	625 (13.60)	917 (17.53)	1891 (15.48)
**Gender**					
	Male	839 (35.08)	1531 (33.32)	1982 (37.88)	4352 (35.62)
	Female	1553 (64.92)	3064 (66.68)	3250 (62.12)	7867 (64.38)
**Medicine category**					
	Rx^a^	2212 (92.48)	4297 (93.52)	4989 (95.35)	11,498 (94.09)
	OTC^b^	84 (3.51)	250 (5.44)	214 (4.09)	548 (4.49)
	No medicine^c^	96 (4.01)	48 (1.04)	29 (0.56)	173 (1.42)
**Total drug cost range (Chinese Yuan^d^)**					
	0-100	957 (40.01)	1742 (37.91)	2048 (39.15)	4747 (38.85)
	100-200	872 (36.46)	1492 (32.47)	1664 (31.80)	4028 (32.97)
	200-300	251 (10.49)	544 (11.84)	481 (9.19)	1276 (10.44)
	>300	312 (13.04)	817 (17.78)	1039 (19.86)	2168 (17.74)

^a^Rx: Receptor X.

^b^OTC: over the counter.

^c^No medicine: patients do not obtain medicine from the capsule clinic and only use the health monitoring system services, such as measuring blood pressure, height, weight, and other basic items.

^d^1 Chinese Yuan=US $0.14.

### Routine Management and Operation

In the context of the United Nations (UN) pilot program, since the capsule clinic concept will be implemented throughout China, its routine management should be considered. The physical medical institutions (community health care stations) that have obtained the “Medical Institution Practicing License” can apply to establish a capsule clinic. The institution and application report should be submitted together to the local health administration department. Doctors from the parent institution provide remote consultations for the associated capsule clinic. Additionally, traditional hospitals (community health care centers), doctors, and the local health administration department are jointly responsible for the routine management. It is important to encourage video surveillance at capsule clinics and to incorporate procedures that ensure the traceability of drugs dispensed automatically. The physical health care center is primarily responsible for not only the unified management of capsule clinics set up by subordinate branches but also the safety of the medical services provided and the quality of the drugs dispensed at the capsule clinic. Drugs dispensed by the intelligent medicine cabinet at the capsule clinic should adhere to the wholesale drug distribution enterprises’ unified procurement guidelines.

## Conclusions and Limitations

With the emergence of capsule clinics, China has a new medical model that can alleviate PHC accessibility problems. Capsule clinics are currently being promoted in every township, village, and community in Ningbo, with the goal of ensuring that every community resident has access to PHC services at their doorstep. The clinics make it convenient for people in remote areas to achieve higher-quality and equitable health resources. In the future, the UN and China will reach consensus on promoting capsule clinics nationwide and become the global template for PHC service delivery. Capsule clinics in remote areas can improve the geographic accessibility for people who lack PHC resources. Capsules can also work well in cities through the use of big data. In many cases and situations, capsule clinics can provide people with the timely PHC services they need, thus reducing financial losses.

**Figure 6:**
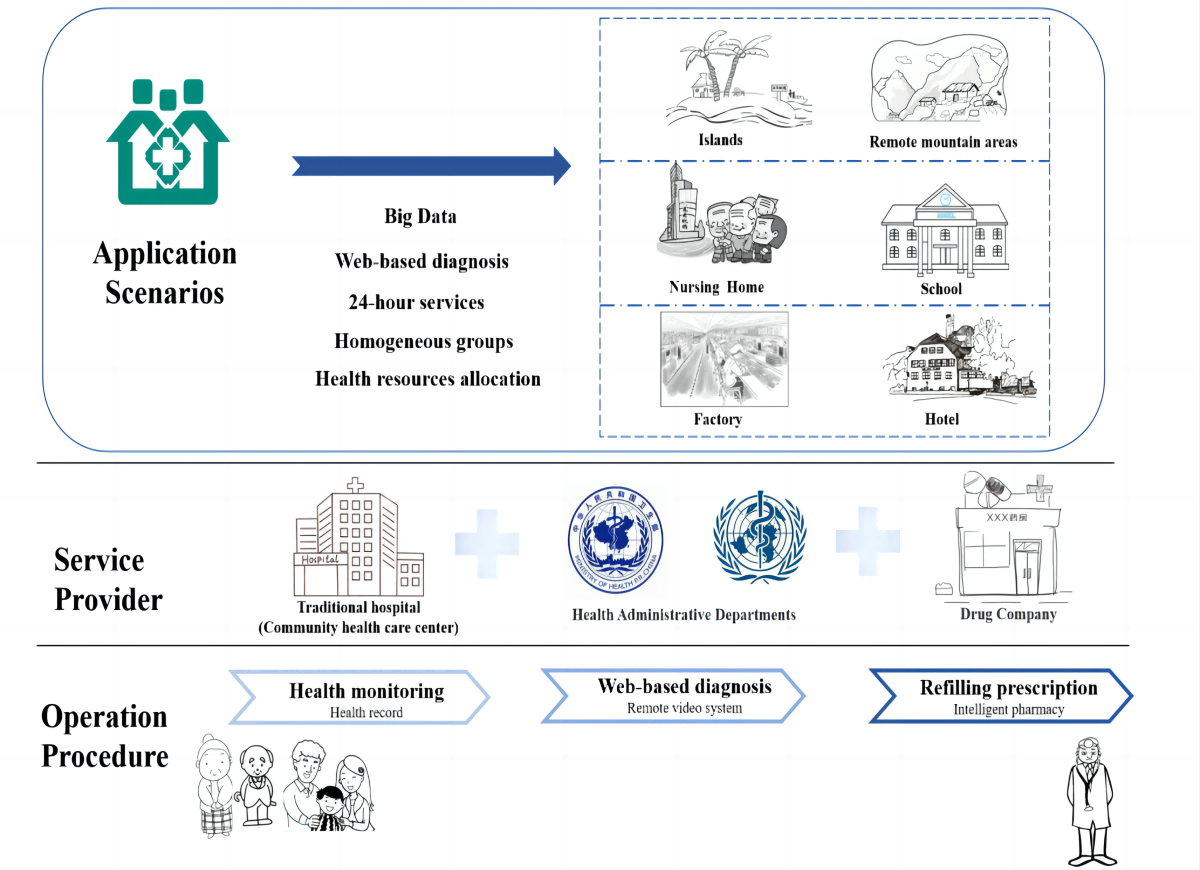
Future application scenarios planned.

Capsule clinics are aimed at people with both common diseases and chronic diseases, and they provide web-based follow-up services, medication disbursement, web-based medical insurance settlement, and more. With the goal of enhanced PHC accessibility, the UN and China intend to place capsule clinics in schools, nursing homes, suburbs, islands, remote mountain areas, and other modern communities. Establishing capsules in these areas can have important effects on many populations. Figure 6 shows that the capsule clinics can be used in the future as follows:

1. Capsule clinics can be placed in remote mountainous areas and islands to increase residents’ access to PHC services. In many cases, low geographic accessibility is a barrier to residents’ access to effective health resources. The capsule clinic can help solve the problem. People in remote areas can receive basic health care services through telehealth. The advantages of capsule clinics, especially in terms of access to essential medicines, are greatly highlighted for these populations.
2. Capsule clinics can use big data to effectively allocate resources, accurately identify regional population characteristics, allocate appropriate PHC resources, and increase investments in the resources needed to meet the various needs of the people. As such, capsule clinics can be placed in homogeneous, crowded places such as schools and nursing homes. As a special place in the community, the school takes the safety of students very seriously, and an on-site capsule clinic can help ensure it.
3. Communities should take advantage of capsule clinics’ 24-hour services. Capsule clinics could be installed in crowded locations with nighttime activity to prevent accidents and, at the same time, offer people timely treatment or access to medication. For example, factories and hotels are key places where things go wrong; an on-site capsule would allow people to seek timely medical help. Doctors are always available digitally to provide reliable advice for seekers.

Of course, the capsule clinic has some limitations. First, data security supervision and drug use safety must be strengthened. Second, the publicity for capsule clinics is not sufficient. People seem to be unaware of this new model or have a wait-and-see attitude. Influenced by traditional health care concepts, numerous patients are more likely to use physical hospitals and are unwilling to use the capsule clinic; consequently, capsule clinic usage is still low. Third, capsule clinics in China are currently limited to urban communities in Ningbo; the single location application and scenario reflect the lack of promotion efforts.

Although the capsule clinic still has some challenges and limitations, it is meaningful in terms of developing convenient and less costly intelligent PHC treatments. People who live in remote areas such as villages and poor mountainous areas far from health care centers face barriers to accessing PHC. Telehealth needs to be enhanced in terms of user experience, and the equipment should be more user-friendly and universal. People are more likely to use smart medical treatment to improve the quality of health services on the basis of the familiar medical treatment mode. Many patients are likely to be driven into poverty by the indirect economic burden of disease. Meanwhile, with higher living standards and enhanced conceptions of health, more and more people are demanding better health products and higher-quality health services. If we are to reach our goal of improving universal health coverage, we must commit to investing in and scaling up proven solutions. Thus, promoting capsule clinics is highly relevant. The intelligent medical industry is still in its infancy, and there is great room for improvement and sufficient potential demand. In the future, the capsule clinic may help solve fundamental imbalances in the distribution of medical resources and contradictions among the growing health care needs of the population.

Countries need to make more and smarter investments in foundational health systems, with an emphasis on PHC, essential services, and marginalized populations. We must make great efforts to ensure equal access to public health services for all.

## Abbreviations

PHC: primary health care
Rx: Receptor X
UN: United Nations
